# Supplementary material for: Role and mechanism of NCAPD3 in promoting malignant behaviors in gastric cancer
Source: Front Pharmacol. 2024 Apr 22;15:1341039. doi: 10.3389/fphar.2024.1341039 (PMC11070777; doi:10.3389/fphar.2024.1341039)
Supplement: Supplementary file 11 [file DataSheet2.ZIP › GSEA/immunologic/my_analysis.Gsea.1599462003817/GSE26030_UNSTIM_VS_RESTIM_TH1_DAY5_POST_POLARIZATION_UP.html]

Details for gene set GSE26030\_UNSTIM\_VS\_RESTIM\_TH1\_DAY5\_POST\_POLARIZATION\_UP[GSEA]

|  || Dataset | filtered\_dataset.sample\_info.cls#WT\_versus\_NCAPD3\_MUT |
| Phenotype | sample\_info.cls#WT\_versus\_NCAPD3\_MUT |
| Upregulated in class | NCAPD3\_MUT |
| GeneSet | GSE26030\_UNSTIM\_VS\_RESTIM\_TH1\_DAY5\_POST\_POLARIZATION\_UP |
| Enrichment Score (ES) | -0.5601027 |
| Normalized Enrichment Score (NES) | -2.4639673 |
| Nominal p-value | 0.0 |
| FDR q-value | 0.013883001 |
| FWER p-Value | 0.138 |
Table: GSEA Results Summary

  

Fig 1: Enrichment plot: GSE26030\_UNSTIM\_VS\_RESTIM\_TH1\_DAY5\_POST\_POLARIZATION\_UP      
 Profile of the Running ES Score & Positions of GeneSet Members on the Rank Ordered List

  

| SYMBOL | TITLE | RANK IN GENE LIST | RANK METRIC SCORE | RUNNING ES | CORE ENRICHMENT || 1 | 4811 | NID1 | 51 | 0.913 | 0.0359 | No |
| 2 | 23054 | NCOA6 | 77 | 0.838 | 0.0845 | No |
| 3 | 7067 | THRA | 976 | -0.392 | -0.5290 | Yes |
| 4 | 91663 | MYADM | 1004 | -0.407 | -0.5160 | Yes |
| 5 | 9308 | CD83 | 1025 | -0.423 | -0.4968 | Yes |
| 6 | 222166 | MTURN | 1030 | -0.429 | -0.4655 | Yes |
| 7 | 8609 | KLF7 | 1071 | -0.457 | -0.4579 | Yes |
| 8 | 604 | BCL6 | 1136 | -0.504 | -0.4638 | Yes |
| 9 | 92014 | SLC25A51 | 1155 | -0.519 | -0.4355 | Yes |
| 10 | 54407 | SLC38A2 | 1182 | -0.553 | -0.4103 | Yes |
| 11 | 64759 | TNS3 | 1197 | -0.572 | -0.3749 | Yes |
| 12 | 7128 | TNFAIP3 | 1198 | -0.573 | -0.3294 | Yes |
| 13 | 157378 | TMEM65 | 1283 | -0.672 | -0.3364 | Yes |
| 14 | 80853 | KDM7A | 1311 | -0.714 | -0.2990 | Yes |
| 15 | 10397 | NDRG1 | 1380 | -0.925 | -0.2744 | Yes |
| 16 | 26959 | HBP1 | 1395 | -1.073 | -0.1992 | Yes |
| 17 | 5055 | SERPINB2 | 1397 | -1.088 | -0.1135 | Yes |
| 18 | 255394 | TCP11L2 | 1408 | -1.538 | 0.0014 | Yes |
Table: GSEA details [plain text format]

  

Fig 2: GSE26030\_UNSTIM\_VS\_RESTIM\_TH1\_DAY5\_POST\_POLARIZATION\_UP      
 Blue-Pink O' Gram in the Space of the Analyzed GeneSet

  

Fig 3: GSE26030\_UNSTIM\_VS\_RESTIM\_TH1\_DAY5\_POST\_POLARIZATION\_UP: Random ES distribution      
 Gene set null distribution of ES for **GSE26030\_UNSTIM\_VS\_RESTIM\_TH1\_DAY5\_POST\_POLARIZATION\_UP**
